# Supplementary material for: Myeloid-Specific Pyruvate-Kinase-Type-M2-Deficient Mice Are Resistant to Acute Lung Injury
Source: Biomedicines. 2022 May 21;10(5):1193. doi: 10.3390/biomedicines10051193 (PMC9138865; doi:10.3390/biomedicines10051193)
Supplement: Supplementary file 1 [file biomedicines-10-01193-s001.zip › biomedicines-1646967-supplementary.pdf]

Supplementary file for

Myeloid-specific pyruvate kinase type M2 deficient mice are resistant to acute lung injury

Xinlei Sun[1,2*], Fujie Shi[1*], Weiran Wang[1], Yunfei Wu[2], Shuang Qu[2], Jing Li[1], Hongwei Liang[2], Ke Zen[1,2¶]

[1]School of Life Sciences, Nanjing University, 163 Xianlin Avenue, Nanjing, Jiangsu, China, 210023; [2]School of Life Science and Technology, China Pharmaceutical University, 639 Longmian Avenue, Nanjing, Jiangsu, 211198, China.

File list:

1. Supplementary Figure S1
2. Supplementary Figure S2
3. Supplementary Figure S3
4. Supplementary Figure S4
5. Supplementary Figure S5
6. Supplementary Figure S6
7. Supplementary Table S1

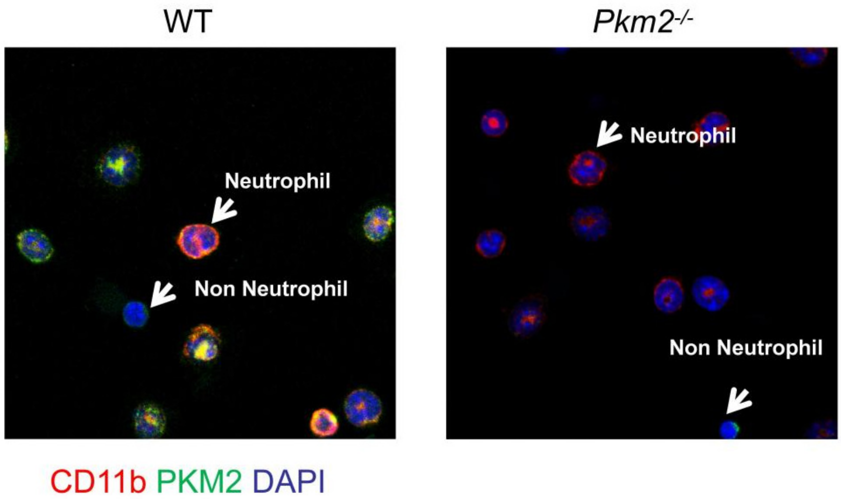

Supplementary Figure S1. Validation of myeloid-specific *Pkm2* knockout in mice by immunofluorescence labeling. Red: CD11b; green: PKM2; blue: DAPI (nuclei).

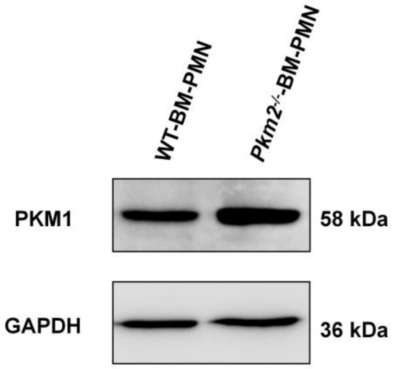

Supplementary Figure S2. myeloid-specific $Pkm2^{-/-}$ mice displayed an increased expression of PKM1 in their myeloid tissues compared to WT mice.

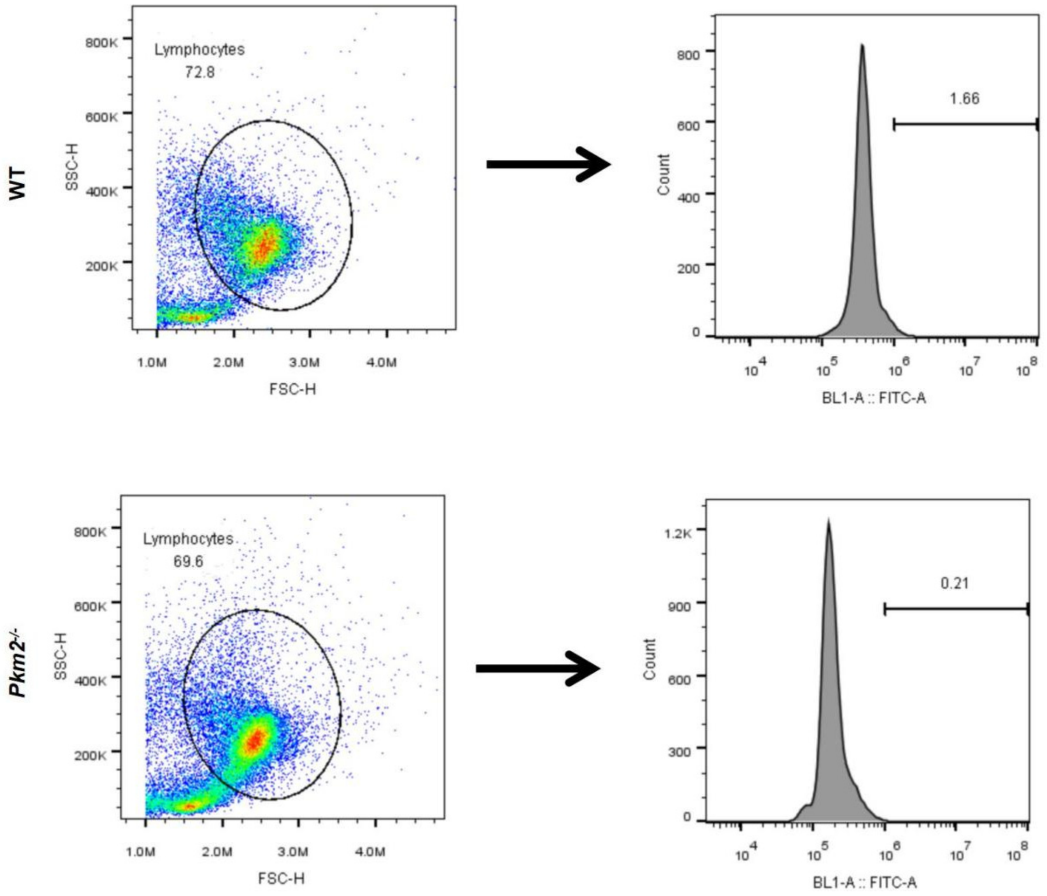

Supplementary Figure S3. Representative plots of flow cytometry data in neutrophils from WT and myeloid-specific *Pkm2*$^{-/-}$ mice.

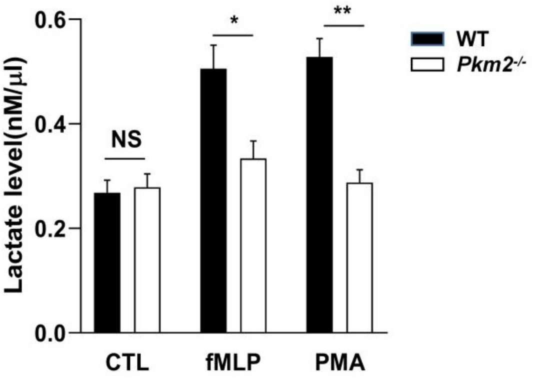

Supplementary Figure S4. Lactate production during PMN degranulation in WT and myeloid-specific *Pkm2*$^{-/-}$ mice. Data are represented as mean ± SEM. *P < 0.05, **P < 0.01.

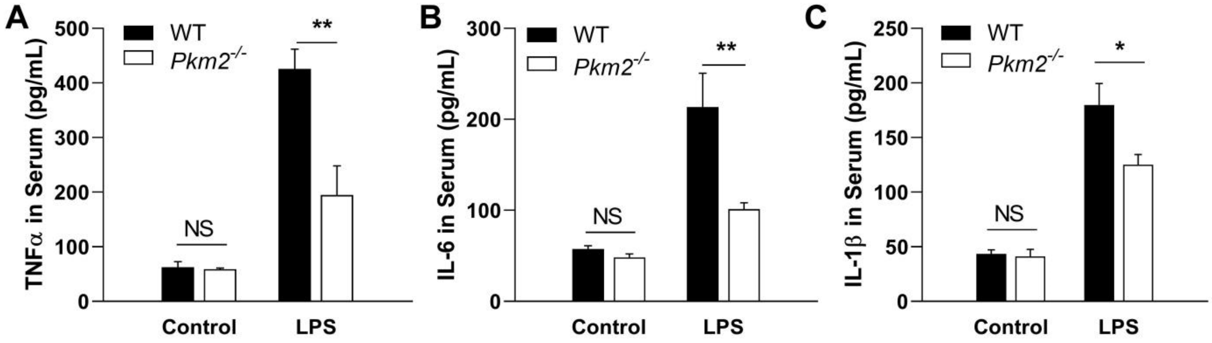

Supplementary Figure S5. IL-1β, IL-6, and TNF-α contents in serum measured using ELISA analysis. Data are represented as mean ± SEM. *P < 0.05, **P < 0.01.

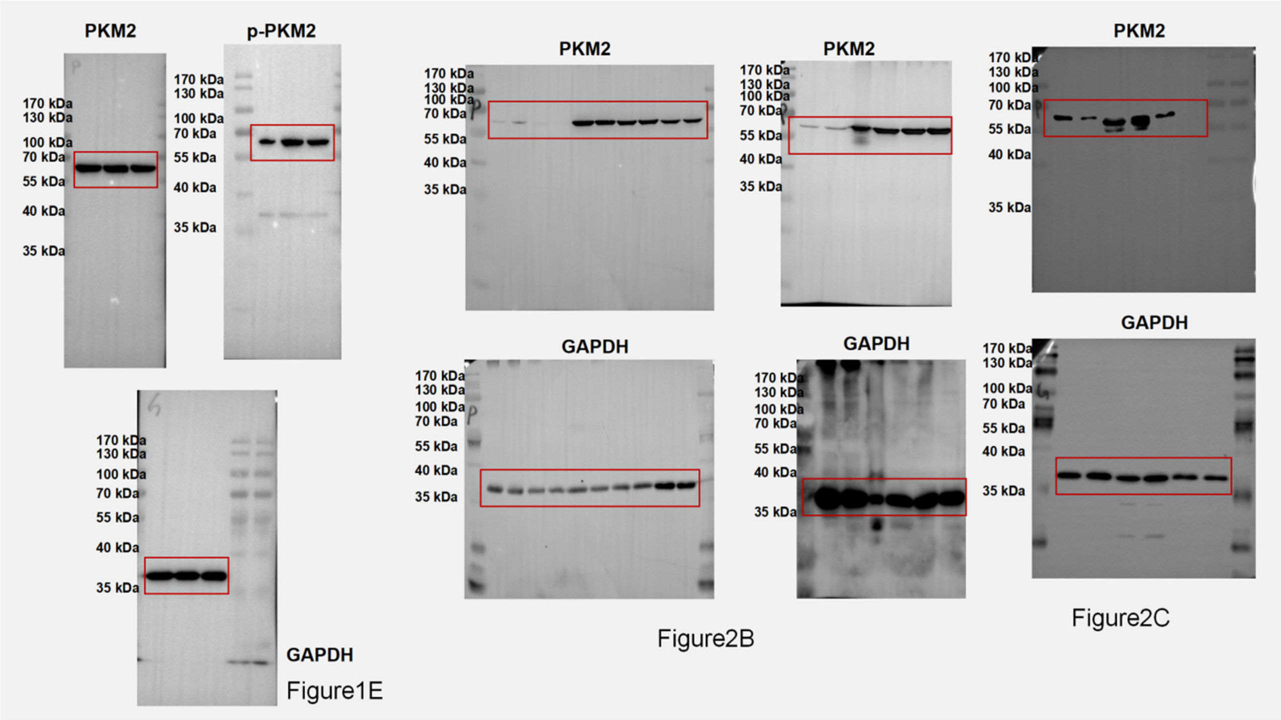

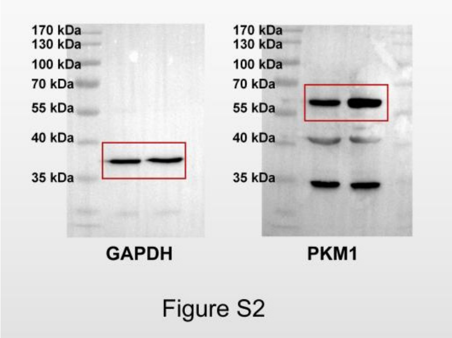

Supplementary Figure S6. Uncropped data of Western blotting.

	brand	catalog	Lot
Human Lactoferrin ELISA	abcam	ab108882	Gr3283761-13
Human MMP9 ELISA	abcam	ab100610	Gr3320948-1
Shikonin	Sigma	S7576	MKBQ3675V
Oleanic acid	Sigma	O5504	SLBG5496V
Serine	Sigma	S4500	SLBN5051V
FBP	Sigma	F6803	SLBH1461V
cytochalasin B	Sigma	C6762	076K4052
fMLP	Sigma	F3506	SLBL4183V
PMA	Sigma	P1585	SLBN5870V
zymosan	Sigma	Z4250	BCBX3813
CD11b	R&D	MAB1124	GQM0317071
P-PKM2	CST	3827S	3
PKM2	CST	4053S	6
MPO	CST	14569S	1
PKM1	Proteintech	15821-1-AP	00021977
GAPDH	ABclonal	AC033	9100033001
anti-CD11b	THERMO	11-0112-41	E08790-1634
LPS	Sigma	L2630	0000102672
Mouse Lactoferrin ELISA	USCN	SEA780Mu	L220418625
Mouse MMP9 ELISA	USCN	SEA553Mu	L220418618
Lactate assay kit	biovision	K607	7L11K06070

Supplementary Table S1. The information of antibodies and reagents.
